# Consensus on the Benefits of the Exsurco Medical Amalgatome SD in the Treatment of Burns and Other Wounds

**Published:** 2019-11-18

**Authors:** Elof Eriksson, Peter Grossman, Tim Pittinger, Chandra Ellis, Justin Gillenwater, Tracee Short

**Affiliations:** ^a^Brigham and Women's Hospital, Harvard Medical School, Boston, Mass; ^b^Grossman Burn Center, West Hills, Calif; ^c^Grossman Burn Center, Bakersfield, Calif; ^d^Grossman Burn Center, Kansas City, Mo; ^e^Akron Children's Hospital, Akron, Ohio; ^f^Bothin Burn Unit, St. Francis, San Francisco, Calif; ^g^Southern California Regional Burn Center at LAC and USC Medical Center, Los Angeles, Calif; ^h^Baton Rouge General Hospital, Baton Rouge, La

**Keywords:** dermatome, excision, debridement, split-thickness skin graft, STSG

**Figure:**
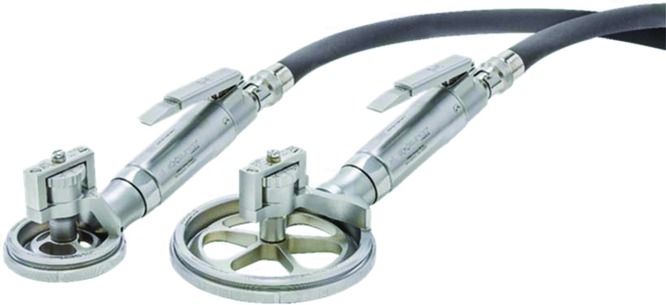
Consensus on the Benefits of the Exsurco Medical Amalgatome SD in the Treatment of Burns and Other Wounds The advisory panel finds that Amalgatome SD provides better speed, precision and patient safety in debridement and skin graft harvesting.

The most common nonpowered surgical instruments for debridement include the Humby knife and the Goulian Weck dermatome, which have remained relatively unchanged since the 1930s.^1^^,^^2^ Clinicians, particularly in the burn and vascular care settings, have experienced the need to have access to a motorized, easy-to-use device for removal of the eschar. Such early excision and grafting will reduce the risk of infection and speed the healing process.^3^ The accuracy in preserving healthy tissue remains a challenge, and it is desirable to be able to use a debriding tool that can remove the eschar more precisely.

In the early 2000s, powered devices that utilize waterjet, cavitating curette, or cavitating nonbladed flat surfaces were introduced for debridement. These technological introductions have proven beneficial and have typically brought benefit to smaller areas of necrosis treated in the outpatient setting.^4^ While helpful in the preservation of healthy tissue and cosmesis, these technological advances do not address the needs when debriding large burns.^5^

To address excisional needs in large burns, surgeons must typically resort to use of the century-old standard of the handheld Humby and Goulian Weck knifes.

In 1949, the first tissue bank was created by Dr George Hyatt for the US Navy to procure donors and recover allograft tissue for use in surgery. The passage of the Uniform Anatomical Gift Act in 1968 was established to maximize opportunities for organ procurement organizations (OPOs) and tissue banks. With the creation of the American Association of Tissue Banks in 1976, the need for more effective and efficient excisional modalities was further emphasized. Driven by other concerns, such as tissue yield, cutting accuracy, and allograft uniformity, these organizations have led the way in the discovery of more precise and effective tissue removal and processing techniques.

In 2013, the Amalgatome MD device (Exsurco Medical, Wakeman, Ohio) was introduced to the OPOs and tissue bank operators as a new circular rotating ring-blade device based on the company's leadership in the protein processing industry and food preparation market. Both OPOs and tissue banks found the circular rotating ring-blade technology provided more controlled and consistent skin graft/allograft procurement as well as increasing tissue yields (Fig 1).

These benefits in precision and yield have made the Amalgatome MD the industry standard for OPOs and tissue bank operators. Nearly 90% of all the split-thickness allograft skin used in the United States has been procured using the Amalgatome MD as seen in Figure 2.

In 2017, the makers of the Amalgatome MD (Exsurco Medical) adapted its technology for surgical use in the burn and hospital trauma centers. The Amalgatome SD shares a few features with traditional dermatomes, such as pneumatically powered capabilities for skin graft recovery (Fig 3). However, the Amalgatome SD has the unique indications to perform both skin grafting and wound debridement/excision. As with the predicate device used in OPOs and tissue banks, the Amalgatome SD features a unique circular rotating ring-blade set in the head of the handpiece, allowing the surgeon to rotate and advance the instrument in multiple directions and titrate cutting depth in a greater range of thicknesses while making an excisional pass.

A published animal study^6^ on the Amalgatome SD demonstrated the device safety and efficacy in viability of collected tissues, speed of healing, and donor site biomechanics. This study demonstrated the unique Amalgatome SD mechanism of action to excise tissue with greater precision and with significantly easier maneuverability than a conventional dermatome that incorporates an oscillating straight blade. The same study determined that the grafts recovered with the Amalgatome SD are very consistent in their thickness.

## METHODS

To develop recommendations on the effective use of the Exsurco Medical Amalgatome SD in the surgical burn setting, the company worked with several of the early adopters of the technology, most of which had used the device more than a year at their respective burn centers.

Exsurco collected their clinical impressions and feedback on the use of the Amalgatome SD and gathered 5 of the users at its inaugural Clinical Advisory Board (CAB) at the American Burn Association annual meeting (Las Vegas, Nev, April 3, 2019).

CAB members include Dr Elof Eriksson, Chair Exsurco CAB, Chief of Plastic Surgery at Brigham and Women's Hospital, Harvard Medical School (ret); Dr Peter Grossman, Director Grossman Burn Centers at West Hills, Calif, Bakersfield, Calif, and Kansas City, Mo; Dr Tim Pittinger, Burn & Pediatric Surgeon, Akron Children's Hospital, Akron, Ohio; Dr Chandra Ellis, Plastic & Burn Surgeon, Bothin Burn Unit at St Francis, San Francisco, Calif; Dr Justin Gillenwater, Director, Southern California Regional Burn Center at LAC and USC Medical Center, Los Angeles, Calif; and Dr Tracee Short, Burn Surgeon, Baton Rouge General Hospital, Baton Rouge, La.

## RESULTS AND DISCUSSION

The consensus of the panel is that the Amalgatome SD represents a significant advancement in debridement in the following areas.

**Figure 4:**
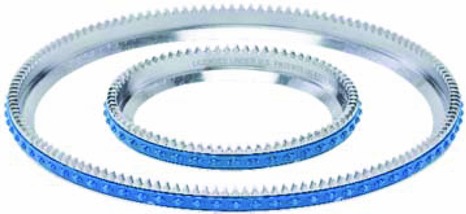
2- and 4-inch excision ring-blades.

**Figure 5:**
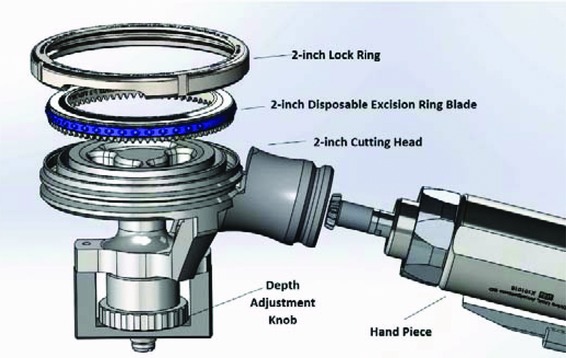
2-inch excision ring-blade assembly into the Amalgatome SD head and handpiece.

**Figure 6:**
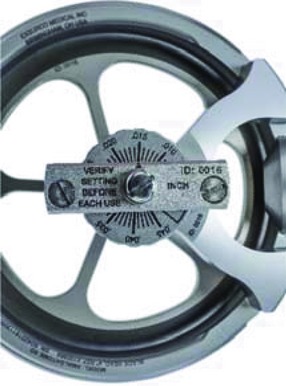
Depth control (top view).

**Figure 7:**
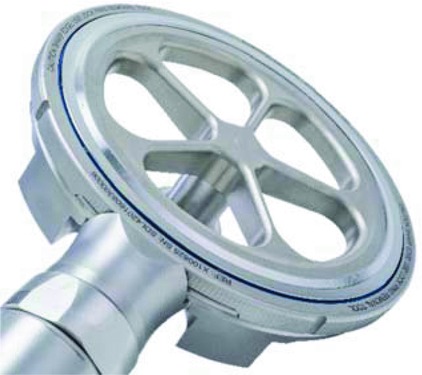
Depth control ring and excision ring-blade (bottom view).

**Figure 8:**
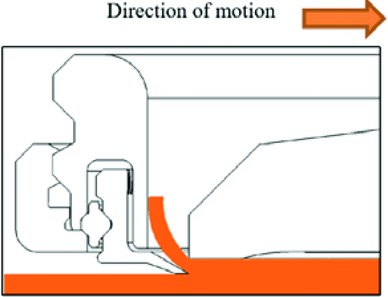
Trailing edge of blade excising skin.

1. The cutting precision is attributed to the rotating blade, which allows for a more precise cut than the competitive oscillating mechanism. It is inherent in the design that the ring prevents the placement of the cutting edge at an angle that would cut too deep and harm the patient. See Figures 4 to 8 to illustrate this unique circular rotating ring-blade set in the head of the handpiece, allowing the surgeon to rotate the instrument in multiple directions and titrate cutting depth while making an excisional pass.
2. The maneuverability of the device is cited for the multidirectional capabilities that enable easy sinuous movements across more challenging areas of the patient such as contoured areas on the body, especially on the abdomen, buttocks, and the head.
3. The ease of use with the device allows debridement procedures to be completed faster and with greater precision than the Humbie or Goulien knives in the operating room setting. CAB members cite ergonomic use and increased patient safety, including the need for less operator force, providing more control over the procedure, resulting in a user experience that is more operator-friendly.
4. Time savings is cited, which can be a critical factor in both the clinical and health economic benefits of the product.

## SUMMARY OF CONSENSUS

1. The Amalgatome SD is an innovative and very useful tool in the treatment of wounds, particularly burns.
2. It greatly improves speed and precision in debridement.
3. It provides better patient safety than other devices for debridement and skin graft harvesting.
4. It is more operator-friendly and with a shorter learning curve than other devices used for debridement and skin graft harvesting.
5. It is particularly useful when harvesting skin grafts from the abdomen, thigh, and scalp.
6. The Amalgatome SD allows more surface area to be either excised or grafted in one procedure.
7. The 4-inch diameter cutting ring is appropriate for many debridement locations, but smaller cutting rings such as the 2-inch ring is preferred for debridement of the face and the hand.

## SUMMARY AND IMPLICATIONS

The clinical advisory panel consensus is that the Amalgatome SD represents significant advancement in the safety and efficacy for debridement, with several panel members finding the device compares favorably overall with competitive products. There is a clinical opportunity for additional research on this device including use for skin grafting.

## Figures and Tables

**Figure 1:**
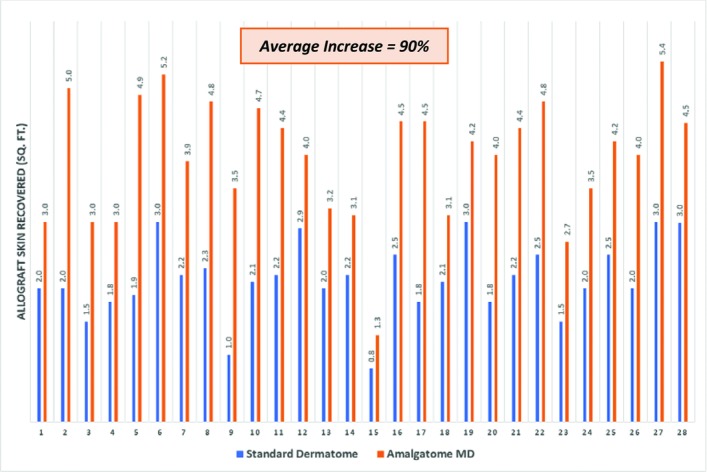
Tissue bank allograft skin yield performance by recovery device.

**Figure 2:**
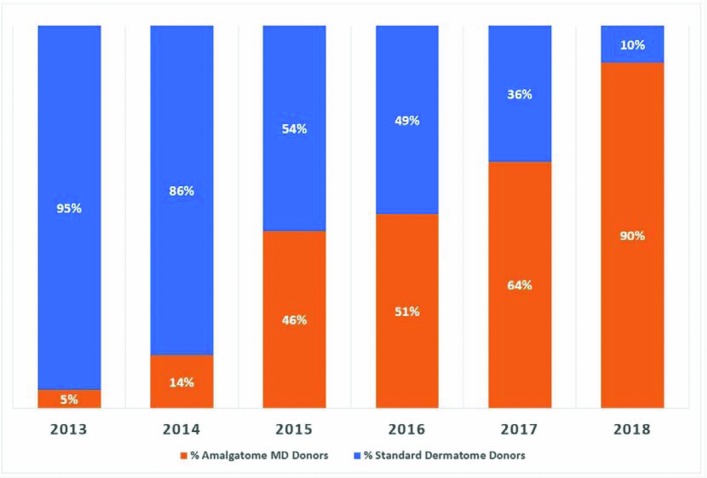
Percentages of market share for split-thickness allograft skin procured by recovery device.

**Figure 3:**
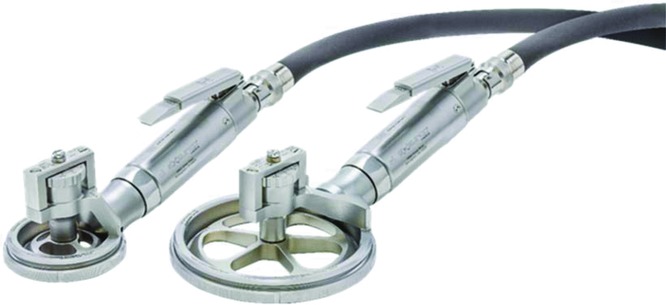
The Amalgatome SD device.
